# HK-ASAP study: protocol for a prospective cohort study on sleep quality, brain reserve and cognitive phenotypes in community-dwelling older adults

**DOI:** 10.1186/s12877-026-07367-0

**Published:** 2026-03-25

**Authors:** Xi Ni, Natalie Shu Yang, Yuk Shan Yuen, Yuqi Gong, Zeyan Li, Xiaoqing Hu, Yixuan Yuan, Hanna Lu

**Affiliations:** 1 Department of Psychiatry, The Chinese University of Hong Kong, Hong Kong SAR, China; 2 Department of Psychology, University of Greifswald, Greifswald, Germany; 3 Department of Linguistics, The Chinese University of Hong Kong, Hong Kong SAR, China; 4 Department of Neuroscience, School of Translational Medicine, Monash University, Melbourne, Australia; 5 Department of Psychology, The University of Hong Kong, Hong Kong SAR, China; 6 Department of Electronic Engineering, The Chinese University of Hong Kong, Hong Kong SAR, China; 7 The Affiliated Brain Hospital of Guangzhou Medical University, Guangzhou, China

**Keywords:** Sleep quality, Brain reserve, Cognition, Healthy longevity, MRI, Brain age, Amyloid beta

## Abstract

**Background:**

The changes of circadian rhythm are very common in older adults, which can lead to decreased quality of life and jeopardize the brain health in the long run. Among all the types of sleep disturbances, poor sleep quality, as a modifiable condition, can greatly affect the daily activities, cognitive functions, quality of life, and even be implicated as a key potential contributing factor in the development of accelerated cognitive decline and prodromal dementia. Consequently, research efforts to understand, and thus potentially model, the effects of sleep quality on cognition and brain during ageing are of great pragmatic values.

**Methods:**

The Hong Kong Cohort of Abnormal Sleep in Ageing Population (HK-ASAP) project is a prospective cohort study recruiting 244 older adults with sleep disturbances. The HK-ASAP study plans to measure sleep quality, brain reserve and cognition at baseline and 2 years. Subjective sleep quality will be assessed by the Pittsburgh Sleep Quality Index (PSQI). Global cognition and domain-specific cognitive functions will be assessed by a comprehensive neuropsychological battery. High-resolution structural magnetic resonance imaging (MRI) data will be acquired and used to quantify the brain reserve, including gray matter volume, cortical thickness and brain age matrices. Saliva samples will be collected for measuring the levels of A*β*40 and A*β*42, and A*β*42/40 ratio. Using the data, we will estimate and compare the sleep quality and its associations with cognition, salivary A*β*, and brain reserve in older adults.

**Discussion:**

The bidirectional relationship between subjective sleep quality and cognitive functions in older adults presents an urgent need to elucidate how poor sleep quality affects cognition and its potential interplay with brain reserve and neuropathological factors. The HK-ASAP study will be a valuable resource for both epidemiological and clinical research, as it will provide fundamental information to identify the individuals at risk of developing cognitive decline. Findings from the HK-ASAP study will inform the development of targeted interventions to enhance sleep quality, cognition, and healthy longevity in older adults.

**Trial registration:**

ClinicalTrials.gov Identifier: NCT06170073 (registered on 01/10/2023).

## Background

Sleep quality is increasingly characterized as an emergent property of brain health in the processes of healthy ageing and neurodegeneration [1, 2]. One of the main reasons is that sleep exhibits profound changes in its structure and endogenous circadian biology in late adulthood [3], which are linked to accelerated cognitive decline and poor health outcomes [1, 4]. Due to the reciprocal relationships between sleep quality and cognitive functions, the clinical condition of cognitive impairments may be aggravated by poor sleep quality [5], and in turn, individuals with poor sleep quality are likely to experience worse cognitive performance and may even be more vulnerable to neurodegenerative diseases [6–9].

In recent years, sleep disturbances have been increasingly recognized as a modifiable risk factor for dementia, particularly Alzheimer’s disease [10]. Notably, a major discovery in this field is that the connection between poor sleep quality and amyloid beta (A*β*) accumulation is essential for cognitive and healthy longevity [10–12]. Consequently, research efforts to understand, and therefore potentially model, the effects of sleep quality on cognition and brain reserve during ageing are of great pragmatic values.

Although a few studies have confirmed the relationship between sleep quality and cognition, a fundamental and unanswered question in this field is: Why do some people have better cognitive maintenance, and why do some people have faster cognitive decline than the others? The status of sleep disturbances, considering as a novel protective factor of cognitive reserve (CR) [13, 14], might provide an interpretation for the discrepancies between individuals with susceptibility to cognitive changes related to ageing. The recent findings indicate that the ones with poor sleep quality may be unable to cope with A*β* accumulation, maintain their cognitive performance and brain reserve in late adulthood than the ones without sleep disturbances. Even at comparable levels of cognitive reserve (e.g., similar education and occupational attainment), poor sleepers appear to experience a more rapid onset of cognitive impairments than good sleepers [15].

Collectively, the present proposal aims to set the baseline characteristics of sleep quality, saliva A*β*, brain reserve and cognition and compare the characteristics of brain structures and cognitive phenotypes in older adults with sleep disturbances by using high-resolution structural MRI scanning and a set of computer-based neurocognitive tests. Thus, we focus our proposal on sleep quality, brain structures and domain-specific cognitive functions as we hypothesize that such explorations will help to identify the relationships between sleep quality, brain reserve and cognitive maintenance in late life. A further aim of this study is to develop a well-characterized, biomarker-confirmed, trial-ready cohort (TRC) to facilitate rapid enrolment into clinical trials utilizing non-pharmacological therapies with subsequent referral pathways for in-clinic evaluation and biomarker confirmation.

## Methods

### Study design and population

This study adopts a prospective cohort design involving community-dwelling older adults with sleep disturbances in Hong Kong. The Hong Kong Cohort of Abnormal Sleep in Ageing Population (HK-ASAP) aims to recruit 244 participants at baseline. The Pittsburgh Sleep Quality Index (PSQI), a widely used instrument in ageing populations, was administered at baseline to assess subjective sleep quality [16, 17]. Inclusion criteria were as follows: 1) Chinese older adults aged ≥ 60 years. 2) Pittsburgh Sleep Quality Index (PSQI) total score ≥ 5. 3) Montreal Cognitive Assessment (MoCA) total score > 22. Exclusion criteria included: 1) Diagnosis of dementia or major neurocognitive disorder. 2) History of major neurological conditions, including stroke, brain tumor, or traumatic brain injury. 3) History of major depression, bipolar disorder or other major psychotic disorders. 4) Contraindications to magnetic resonance imaging (MRI) scanning.

### Data collection and measurement

For recruitment, we use a combination of referral methods that builds trust and addresses clear and detailed study aims and logistics. The methods include: 1) placing study flyers at elderly centres, 2) publishing advertisements on local newspaper and social media (e.g., Facebook) to reach potential participants. Older adults residing in the New Territories, Kowloon and Hong Kong Island will be recruited and undergo a pre-screening assessment of subjective sleep quality and cognitive functions at Tai Po Hospital. During screening, participants will go through a process that collects information on key variables, including PSQI, demographic information etc. Moreover, participant’s current residential address and whether one is undergone a disease treatment will also be recorded to guarantee our target population to be community dwelling older adults and exclude individuals reside in a nursing home, long-term care facility, assisted living facility, or other institutional care setting. As part of the study design, we match the participants in two groups based on these variables to ensure comparability and minimize bias in the analysis. Baseline data collection for HK-ASAP study began in October 2023 and will continue until the target sample for this study has been completed. We will collect basic demographic information, occupation and leisure activity data, subjective sleep quality, health-related data, cognitive functions and imaging data on the various dimensions of brain reserve using the measures described here.

### Sleep and health-related data

Individual’s subjective sleep quality is assessed using the Pittsburgh Sleep Quality Index (PSQI), a validated 19-item self-reported questionnaire [16, 17]. PSQI items could be grouped into seven subcomponent scores representing the dimensions of subjective sleep data: sleep quality (C1), sleep latency (C2), sleep duration (C3), sleep efficiency (C4), sleep disturbances (C5), sleep medication use (C6), and daytime dysfunction (C7). Each subcomponent score ranges from 0 to 3, with higher scores reflecting poorer sleep quality. Health-related data includes the status of smoking, alcohol use, drinking tea or coffee, neurovascular risk factors, lifestyle activities, and the conditions of chronic diseases.

### Salivary A*β*40 and A*β*42 data

Saliva samples are collected at a consistent time of day to avoid circadian effects and will be kept on ice during the collection. The levels of A*β*40 and A*β*42 in the saliva samples will be quantified by enzyme-linked immunosorbent assay (ELISA)-type assays (Aurin Biotech, Inc.) [18]. All the ELISA assays are conducted within the Prince of Wales Hospital. Salivary A*β*40 and A*β*42 level, and the ratio of A*β*42 and A*β*40 are used as neuropathological markers in this study.

### Cognitive data

Global cognition is measured using the Montreal Cognitive Assessment (MoCA). MoCA is a 30-point neuropsychological test assessing primary cognitive domains, including attention, visuospatial ability, executive function, short-term memory, and orientation [19]. The Hong Kong version MoCA (HK-MoCA) has been validated in the local community-based population and shows sensitivity to changes in global cognition in Alzheimer’s disease and vascular dementia [20]. Higher MoCA score indicates better global cognition.

Complex attention, including alerting, orientation and executive control, is assessed by the Attention Network Test (ANT), run on E-Prime 3.0. The ANT employs a 4 (cue type: no, double, center, spatial) × 3 (flanker type: neutral, congruent, incongruent) factorial design [21]. In a standard trial, a randomized fixation period (400 ~ 1600 ms) is followed by a brief warning cue (100 ms). The target is a central arrow appearing above or below fixation that presented with flanking arrows on each side. There are two measures used to evaluate the three components of complex attention, which are accuracy and reaction time (RT). As a quality control measure for ANT data, accuracy quantifies the degree of decision correctness. Any experimental condition with an accuracy below 70% fails the quality standard. RT, measured in milliseconds (ms), indicates how quickly a stimulus is processed. The scores of alerting, orientation and executive control are calculated based on the reaction time (RT) of different cues and flanker types [21]. The efficiency of alerting is calculated by subtracting the RT of center cue from the RT of no cue. The efficiency of orientation is calculated by subtracting the RT of spatial cue from the RT of center cue. The efficiency of executive control is calculated by subtracting the RT of congruent flanker from the RT of incongruent flanker.

Working memory and learning function are assessed using word-list learning test (WLLT) [22]. This cognitive test consists of ten semantically unrelated Chinese words. The procedure includes three consecutive free-recall trials (assessing immediate recall and learning ability), followed by a 20-minute delayed recall trial to prevent recency effects, and concludes with a yes/no recognition test. The raw scores from the number of words recalled in each phase are used to calculate proxies for learning and memory function. The numbers of the correct recalled words are used as proxies of working memory function. The proxies of learning function included: 1) Initial leaning: the number of words recalled on trial 1. 2) Best learning: the number of words recalled on trial 3. 3) Total learning is calculated as the sum of the number correctly recalled from trial1 to trial 3.

Executive function is evaluated using the Category Verbal Fluency Test (CVFT). For each of three 60-second trials, participants are asked to verbally generate as many unique words as possible belonging to a specified semantic category: animals, fruits, or vegetables [23]. The total number of correct words generated across all categories are used as the primary measure of executive function. The higher number of correct words indicates better executive function.

### Structural MRI data

#### Data acquisition

For collecting high-quality neuroimaging data, structural magnetic resonance imaging (MRI) data are collected at the Prince of Wales Hospital using a 3.0 Tesla Siemens MAGNETOM Prisma MRI scanner. T1-weighted magnetization prepared rapid gradient echo (MPRAGE) sequence is used to optimize the grey-white contrast, with the following parameters [24]: axial acquisition with a 256 × 256 × 192 matrix, thickness = 1 mm, no gap, field of view (FOV) = 230 mm, repetition time (TR) = 2070 ms, echo time (TE) = 3.93 ms, flip angle = 15^◦^. The sequence yields high quality isotropic images with the voxel size of 1 mm ×1 mm × 1 mm.

#### Proxies of brain reserve

Brain reserve (BR) is a concept in ageing neuroscience that refers to the neurobiological or structural capital of the brain, such as the number of neurons, synapses, brain volume, or other anatomical features that provide a buffer against neuropathology or age-related cognitive decline [25, 26]. Moreover, as a complex and multidimensional construct, BR can hardly be directly captured by a single neuroimaging measure.

#### Traditional measures for BR

Based on structural MRI scans, surface-based morphometric analysis (SBM) will be conducted by BrainSuite 23 (http://brainsuite.org/). The image processing methods contain a series of steps: 1) head motion correction; 2) image intensity normalization; 3) removal of the non-brain voxels; 4) segmentation of gray matter (GM), white matter (WM) and cerebrospinal fluid (CSF); (5) tessellation of GM/WM boundary, and topology correction. After processing, the commonly used quantitative measures of BR include the volumes of GM and WM, and cortical thickness [27–29].

#### Brain age matrices

Beyond traditional measures of BR, brain age is typically estimated by pre-training machine learning models to predict chronological age from the MRI scans of healthy adults. These models are then applied to new participants to derive an estimated brain age. The discrepancy between estimated brain age and chronological age, termed as the brain-predicted age difference (brain-PAD), captures individual’s deviations from normative ageing trajectories [30]. Brain age matrices include two components: estimated brain age and brain-PAD, which can serve as imaging phenotypes in the diagnosis and prognosis of age-related neurodegenerative diseases [31–33].

Firstly, a pre-trained brain age model is built using the structural MRI data from the Cambridge Centre for Ageing and Neuroscience (Cam-CAN) project (*N* = 609, aged from 18 to 90 years) [34]. Whole-brain morphometric features from this cohort are applied to train a support vector regression (SVR) model with a linear kernel in MATLAB (fitrsvm function). The brain age model is trained and internally validated through 10-fold cross-validation on the entire Cam-CAN dataset. Subsequently, participants from HK-ASAP study will serve as an independent testing cohort to compute brain age matrices and evaluate model generalizability in a local ageing population.

### Follow-up and retention

At baseline, we collect participants’ names, phone numbers, addresses, dates of birth, marital status, handiness and health-related behaviours, including the habits of using alcohol and tobacco, drinking tea and coffee, and the status of vascular risk factors and chronic diseases and use these variables as covariates. Subjective sleep quality, global cognition, domain-specific cognitive function, the proxies of brain reserve and salivary A*β* levels are collected at baseline and 2-year follow-up and used as outcome measures. Participants are paid HK$ 100 for travel fee at each time point of assessment.

### Sample size estimation and power analysis

Study has shown that sustained high insomnia symptoms are associated with accelerated global cognitive decline, while chronic insomnia confers a 40% increased hazard of incident cognitive impairment and faster global decline [35]. Moreover, comparing to global cognition (approximately 0.10–0.20 SD) [36], domain-specific cognitions are more vulnerable to the persistent sleep disruption. Executive function and memory, in particular, have shown larger effects in higher-risk or insomnia-affected subgroups, generally in the range of approximately 0.25–0.35 SD [37, 38]. Persistent insomnia has also been associated with accelerated cognitive decline and increased risk of incident cognitive impairment [35, 39]. Given that this study targets a sleep-disturbance–enriched population (PSQI ≥ 5) and prioritizes domain-specific cognitive change, we conservatively assumed a standardized effect size of 0.30 SD over 2 years. With a two-sided *α* of 0.05 and 80% power, and around 5–6% attrition, the target sample size was set at 260 (final *N* = 244) participants.

### Statistical analysis

Descriptive statistics will be computed to describe the baseline characteristics of the participants (e.g., age, sex, education). Normally distributed variables will be described by the mean and standard deviation. We will examine the association between sleep quality, cognitive functions, brain reserve, and salivary A*β* levels using baseline data. Firstly, the PSQI total score will be used as continuous variable for detecting the correlations with health features, cognition, brain age matrices and saliva A*β* levels in general. Secondly, to define the severity of subjective sleep quality, we will use the total score of PSQI as existing literature described, that is mild sleep disturbances as PSQI total score between 5 and 10, and moderate-to-severe sleep disturbances as PSQI total score > 10 [40]. In the analysis, we will look for correlations between the components of PSQI. We also compare the brain reserve, cognitive functions and salivary A*β* levels between the older adults with mild and moderate-to-severe sleep disturbances.

Multivariable logistic regression models, incorporating baseline and follow-up data, will assess whether the longitudinal changes in subjective sleep quality predict neuropathological progression, cognitive maintenance, or the conversion to cognitive impairments or dementia. Meanwhile, multiple linear regression models will be used to investigate the relationships between salivary A*β* levels, brain reserve and cognitive maintenance. We will also construct the Cox regression models to test the effects of subjective sleep quality on brain reserve, cognitive functions and healthy longevity. The events used in Cox regression include incident dementia, major functional decline, or death, depending on the available follow up data. All the analyses will be undertaken using STATA/SE version 14; 2-sided *p* < 0.05 is considered statistically significant.

### Dissemination plan

Study results will also be disseminated to study participants, their families and community partners who work with this population through a variety of methods. We will also disseminate the results through academic journals to reach other researchers focused on sleep, cognition and brain health.

## Discussion

This observational cohort study will contribute fundamental knowledge on the dynamic relationships between sleep quality, brain reserve, cognition and A*β* in ageing population. In the past two decades, there are several high-profile, longitudinal case-control cohort studies that study the relationship between sleep and ageing. For example, the Rotterdam Study focuses on the causes and outcomes of age-related diseases [41]. Based on the sleep quality measured by PSQI, this cohort has shown that both short and long sleep durations are associated with increased risk of dementia and stroke [42]. The Sleep Heart Health Study (SHHS) has provided crucial data linking poor sleep to accelerated cognitive decline, brain structural changes, and increased risk of dementia in older adults [43]. The HK-ASAP study focuses on the cycle of “sleep quality-cognition-A*β*-brain” in the context of ageing, which can help identify imaging (i.e., brain reserve) and cognitive phenotypes associated with sleep disturbances and poor health outcomes at individual level. By measuring sleep quality, health-related features, domain-specific cognition functions and their relations to saliva A*β* and brain reserve, we hope that the HK-ASAP study can be a trial-ready cohort (TRC) to identify the targeted individuals for non-pharmacological interventions to enhance sleep quality and reduce the risk of developing dementia. A 2-year longitudinal follow-up assessment will also allow us to evaluate the impact of subjective sleep quality on A*β*, cognitive functions and brain reserve over time. Furthermore, this study will also address the paucity of ageing research on how subjective sleep quality interacts with neuroimaging and cognitive phenotypes.

### Strengths and limitations

The HK-ASAP study will add a significant amount of data regarding brain reserve, cognition and A*β* in older adults with sleep disturbances, and will address some of the prior weakness in this field. It will be the first study to date on the topic of sleep quality, brain reserve, A*β* and cognition in Asia. The proxies of brain reserve, including both the surface-based morphometry and novel brain age matrices, are multiscale quantitative brain measures. The HK-ASAP study will offer an in-depth understanding of the complex, dynamic relationships between sleep disturbances, A*β*, brain reserve and cognitive maintenance, providing value research data for scientists and clinicians. The findings from HK-ASAP study will provide fundamental evidence for identifying the individuals at risk of developing cognitive impairments and enable clinicians to develop interventions to improve the sleep quality, cognition and healthy longevity in ageing populations.

The HK-ASAP’s limitations include a potential lack of generalizability of findings to certain communities and groups because of the selection bias. Commonly, older adults who lived in public rental houses tend to have sleep disturbances than the general ageing population with more reserve (e.g., socioeconomical status, housing conditions) in Hong Kong. The targeted recruitment efforts, including local newspaper advertisement, will be made to ensure a diverse group of participants with different cognitive reserve can be enrolled. Besides, health conditions or the comorbidities of chronic diseases are very common in older adults, which could affect sleep quality and cognition as well. In HK-ASAP study, we collect the health-related information, including habit of drinking and smoking, chronic diseases, medication use, and use the information as controlled factors at baseline. Beside of bias, this study still has some other limitations. First, brain reserve is a complex and multidimensional concept. Therefore, structural MRI measures and brain-age estimates used in this study should therefore be interpreted as proxy indicators intended to approximate reserve- and maintenance-related mechanisms. Future work is needed to further refine and validate imaging-based measures of brain reserve. Second, although saliva-based testing of A*β* is a non-invasive and cost-effective research approach for early-stage AD, it faces significant limitations. Specifically, the analytical variability of salivary A*β* measurements is relatively higher compared to CSF and plasma sample due to lower biomarker concentrations, greater influence of oral contamination, and less standardized collection protocols [44]. Due to the lack of genetic information in this cohort, we are unable to account for genetic risk factors in our analyses. The genetic information, especially APOE genotype, may modify both sleep effects and brain ageing trajectories [45, 46]. Future studies incorporating APOE *ε*4 carrier status and polygenic risk scores for AD would provide valuable insights into gene-environment interactions between sleep quality and brain health.

## Data Availability

The datasets used and/or analysed during HK-ASAP study will be available from the corresponding author on reasonable request.

## Abbreviations

Aβ: Amyloid beta
AD: Alzheimer’s disease
ANT: Attention network test
Brain-PAD: Brain-predicted age difference
CR: Cognitive reserve
CVFT: Category Verbal Fluency Test
ELISA: Enzyme-linked immunosorbent assay
GM: Gray matter
HK-ASAP: Hong Kong Cohort of Abnormal Sleep in Ageing Population
MoCA: Montreal cognitive assessment
MPRAGE: Magnetization prepared rapid gradient echo
MRI: Magnetic resonance imaging
OR: Odds ratio
PSQI: Pittsburgh Sleep Quality Index
TRC: Trial-ready cohort
WLLT: Word-list learning test
WM: White matter
